# Supplementary material for: Visually guided homing of bumblebees in ambiguous situations: A behavioural and modelling study
Source: PLoS Comput Biol. 2020 Oct 13;16(10):e1008272. doi: 10.1371/journal.pcbi.1008272 (PMC7553325; doi:10.1371/journal.pcbi.1008272)
Supplement: S1 Fig — For each condition, we represented 3 trajectories examples. The examples were selected based on the amount of time the bees spend searching. Each trajectory is colour coded. The longest flight trajectory is in blue, the shortest in red and the average length trajectory for this condition ín black. (PDF) [file pcbi.1008272.s001.pdf]

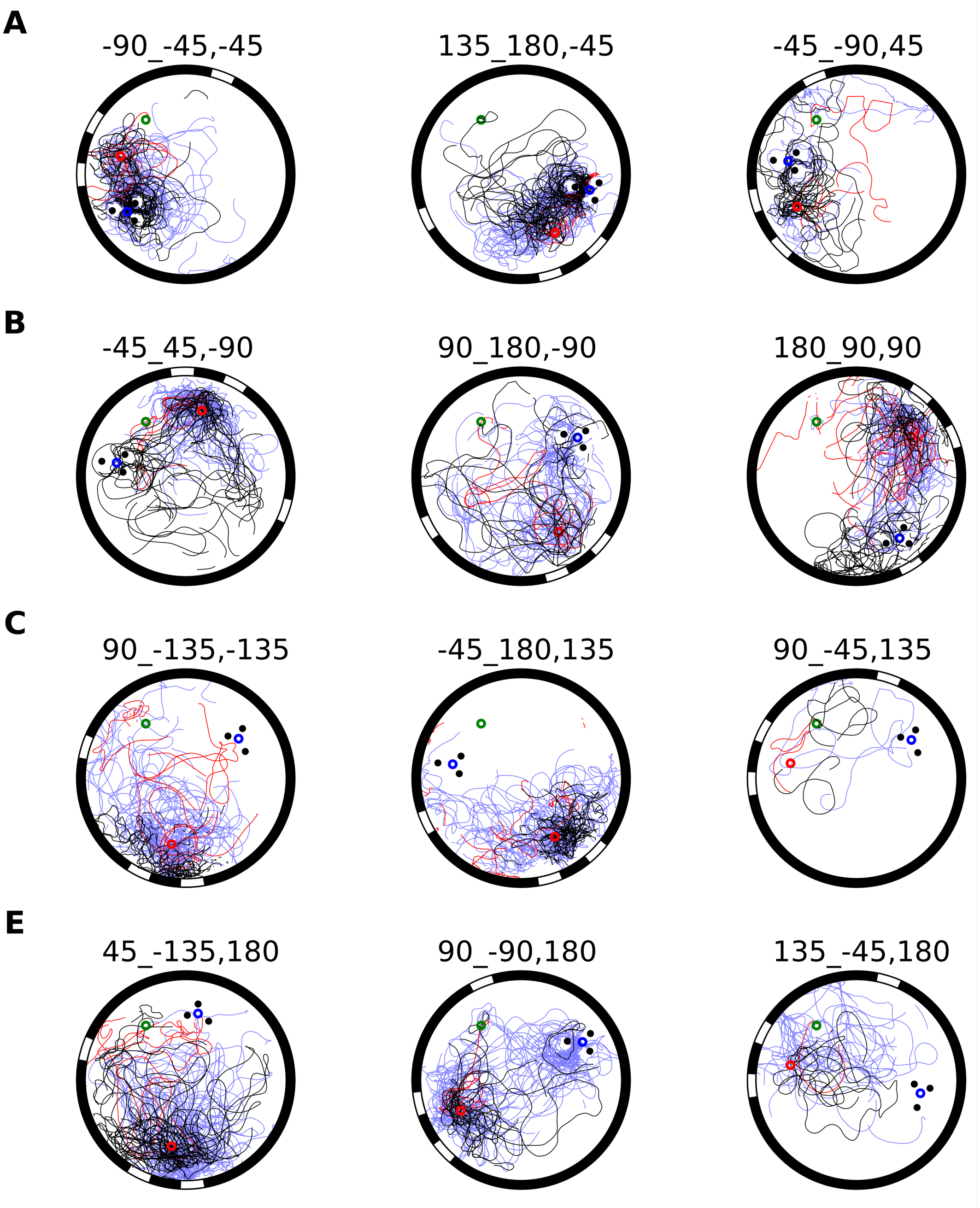

S1 Fig Trajectories examples and illustration of the behavioural variability. For each condition, we represented 3 trajectories examples. The examples were selected based on the amount of time the bees spend searching. Each trajectory is color coded as follow: The bumblebee which spend the most time in blue, the less time in red and the bumblebee spending the average amount of time in black.
