# Supplementary material for: Visually guided homing of bumblebees in ambiguous situations: A behavioural and modelling study
Source: PLoS Comput Biol. 2020 Oct 13;16(10):e1008272. doi: 10.1371/journal.pcbi.1008272 (PMC7553325; doi:10.1371/journal.pcbi.1008272)
Supplement: S2 Fig — The ALV model is not affected by the change in altitude since it uses the exact position of the cues in the 2D plane. (PDF) [file pcbi.1008272.s002.pdf]

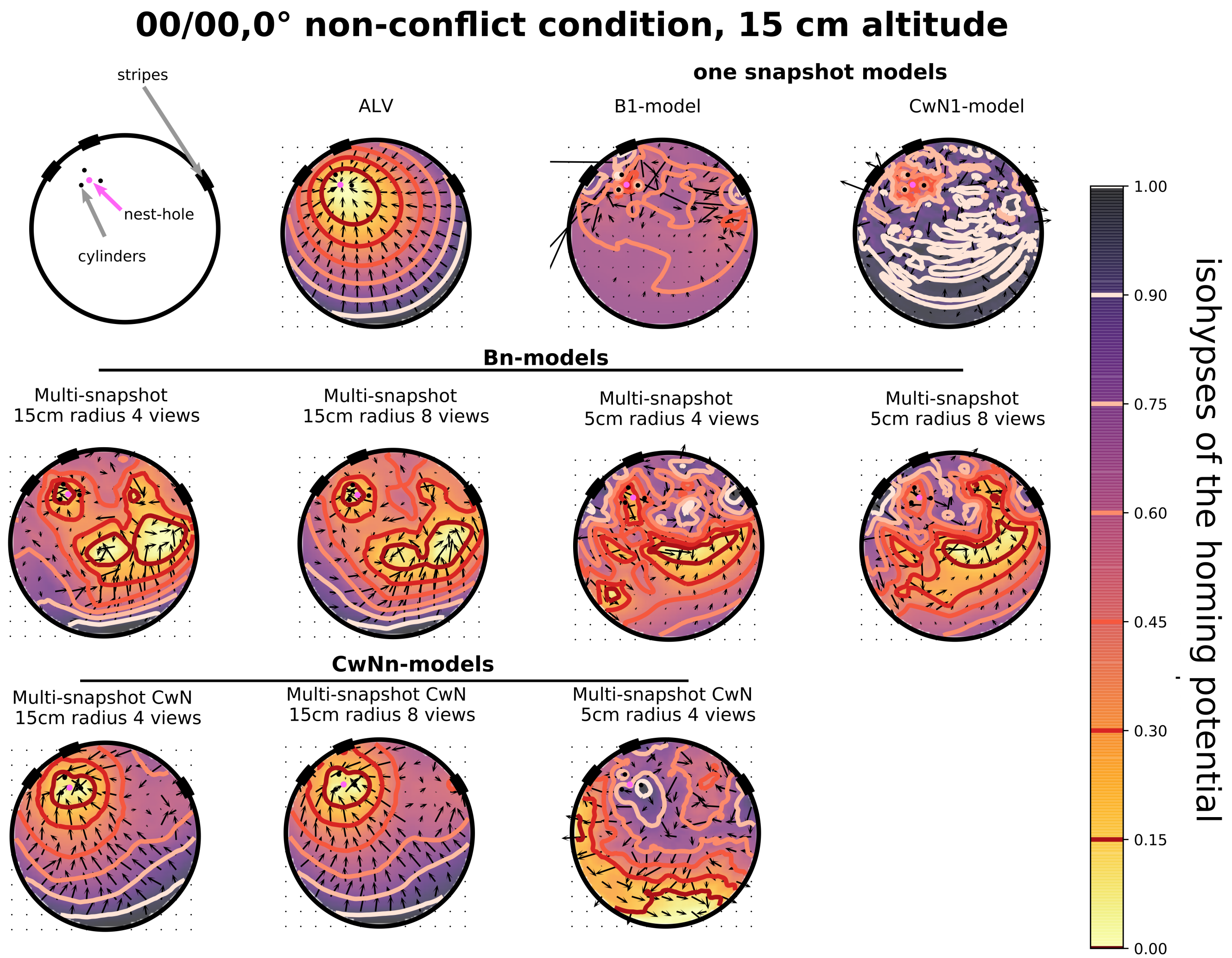

S2 Fig Homing potential of all different models during non-conflict situation at 15cm elevation. The ALV model is not affected by the change in altitude since this one use the exact position of the cues in the 2D plane.
