# Supplementary material for: Visually guided homing of bumblebees in ambiguous situations: A behavioural and modelling study
Source: PLoS Comput Biol. 2020 Oct 13;16(10):e1008272. doi: 10.1371/journal.pcbi.1008272 (PMC7553325; doi:10.1371/journal.pcbi.1008272)
Supplement: S3 Fig — Each subplot represents the confusion matrix output for each model during tested conflict conditions, from left to right: ALV, B8-model, and the CwN4-model. Each title informs the tested condition followed by its F1-score. The colours describe the correct predictions: the true negatives in orange and the true positives in yellow, while the failure of the model predictions are: false positives in purple and the false negatives in dark blue. (PDF) [file pcbi.1008272.s003.pdf]

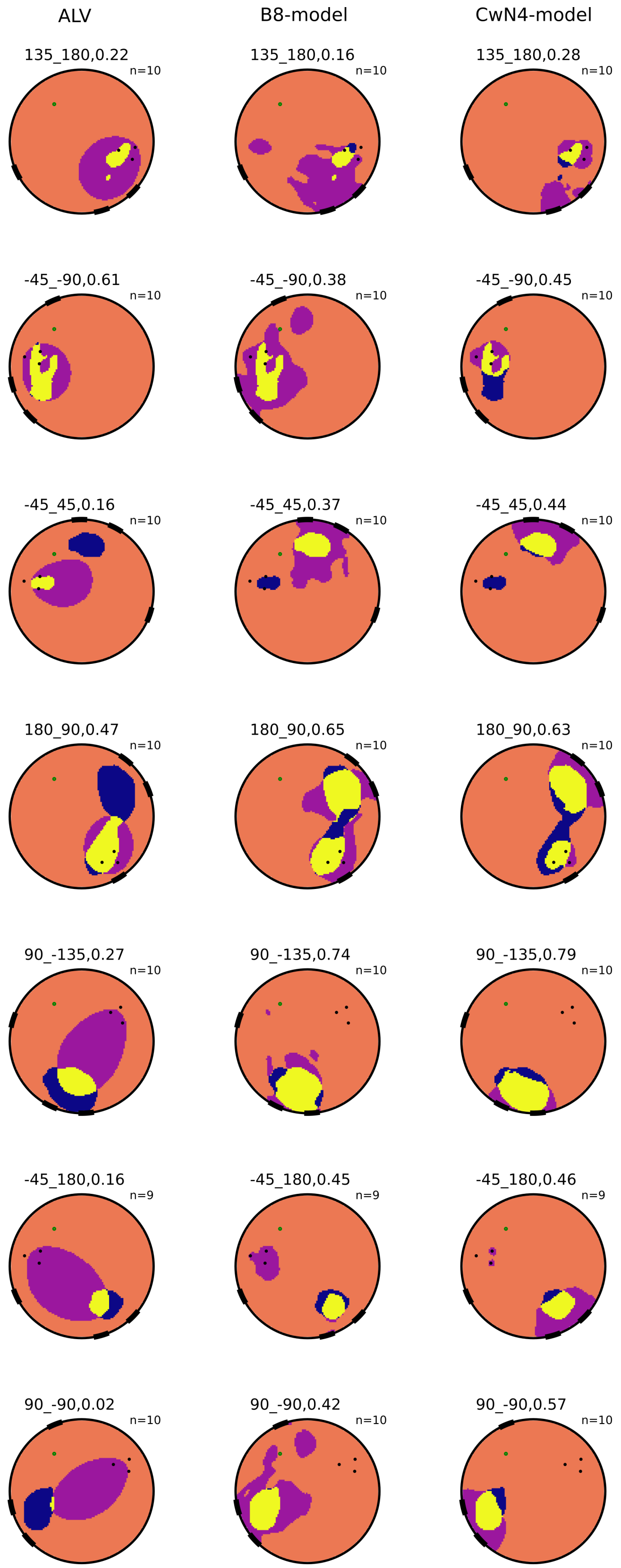

S3 Fig 2D representation of the confusion matrix values for each conflict conditions.Each subplots represents the confusion matrix output for each model during tested conflict conditions, from left to right, first column ALV, second column B8-model, and third column the CwN4-model. Each title informs the tested condition followed by its F1-score. The colours describe the correct predictions: the true negatives in orange and the True positives in yellow while the failure of the models prediction are: false positives in purple and the false negatives in dark blue.
