# Supplementary material for: Visually guided homing of bumblebees in ambiguous situations: A behavioural and modelling study
Source: PLoS Comput Biol. 2020 Oct 13;16(10):e1008272. doi: 10.1371/journal.pcbi.1008272 (PMC7553325; doi:10.1371/journal.pcbi.1008272)
Supplement: S4 Fig — Each plots represents for each condition the F1-score depending on the selected isohypse from 0.1, pink, to 0.29, light brown. A Dunn’s Post-hoc test following the Kruskall-Wallis test was performed on each condition: the adjusted significance values are represented when significant. The significance levels are coded as follow: p<0.5 *, p<0.1 **, p<0.01 **, p<0.001 ***, p<0.0001 ****. (PDF) [file pcbi.1008272.s004.pdf]

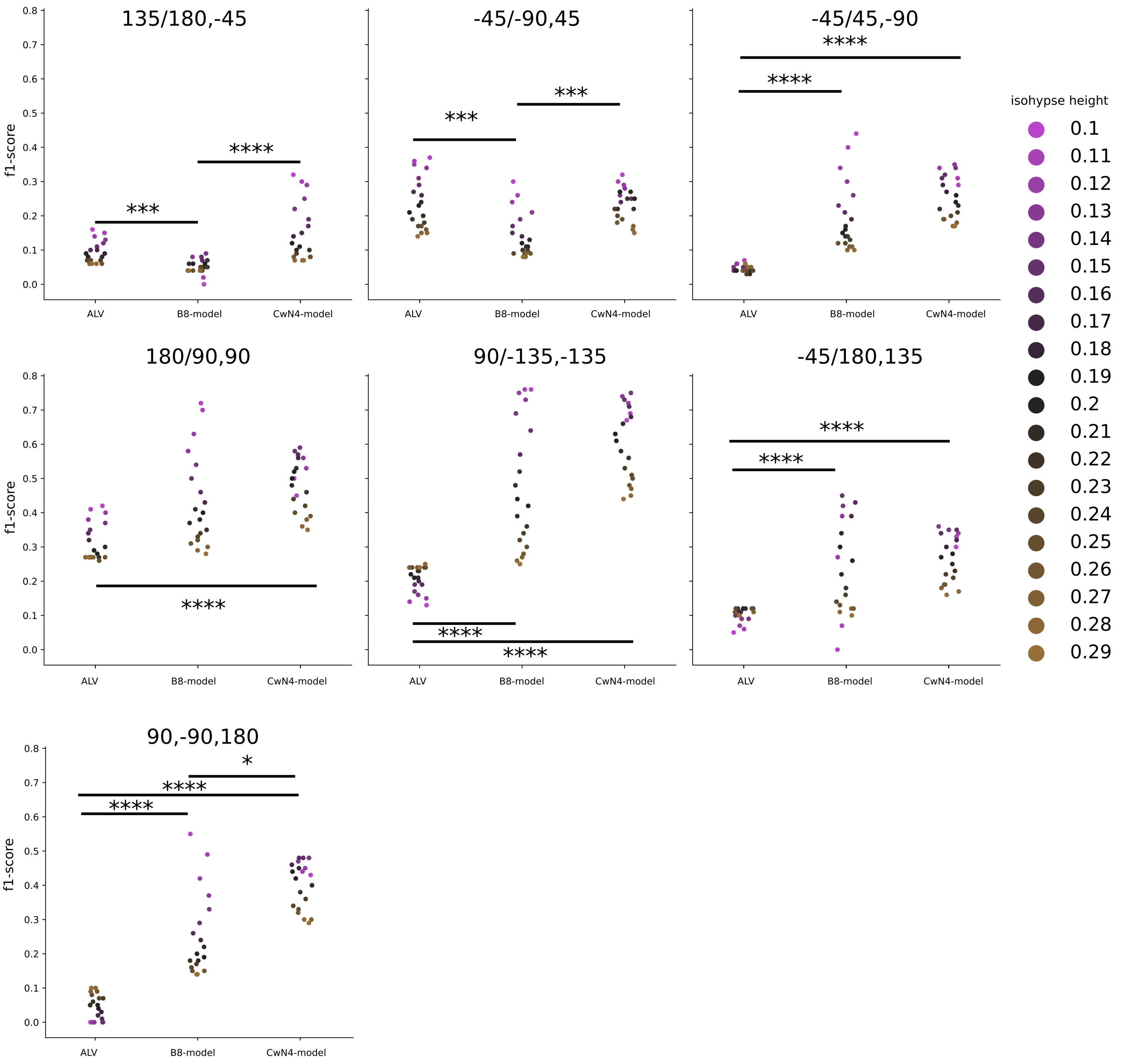

S4 Fig F1-score when varying the model prediction isohypse. Each plots represents for each condition the F1-score depending on the selected isohypse from 0.1, pink, to 0.29 ,light brown. A Dunn's Post -hoc test following the Kruskall-wallis test was perform on each conditions: the adjusted significance values are represented when significant. The significance levels are coded as follow: p<0.5 *, p<0.1 **, p<0.01 **,p<0.001 ***, p<0.0001 ****.
