# Supplementary material for: Visually guided homing of bumblebees in ambiguous situations: A behavioural and modelling study
Source: PLoS Comput Biol. 2020 Oct 13;16(10):e1008272. doi: 10.1371/journal.pcbi.1008272 (PMC7553325; doi:10.1371/journal.pcbi.1008272)
Supplement: S5 Fig — The 4 views represented as an equirectangular projection is overlaid with the corresponding rotIDF function with the current view as in Fig 2B. (PDF) [file pcbi.1008272.s005.pdf]

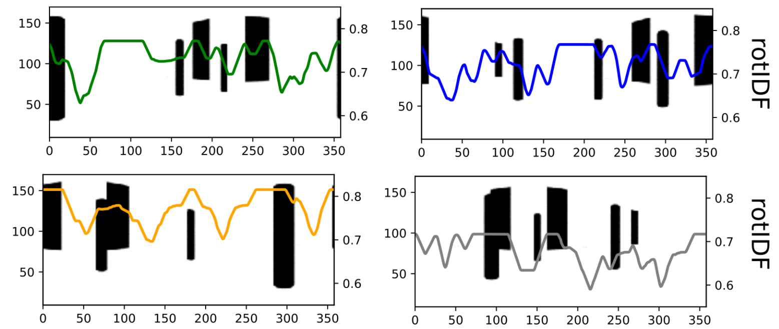

S5 Fig The set of views S for the multi-snapshot brightness model taken at 5cm from nest hole. The 4 views represented as an equirectangular projection constituting the set of view, is overlaid with their own rotIDF function with the current view as in Fig 2B.
