# Supplementary material for: Visually guided homing of bumblebees in ambiguous situations: A behavioural and modelling study
Source: PLoS Comput Biol. 2020 Oct 13;16(10):e1008272. doi: 10.1371/journal.pcbi.1008272 (PMC7553325; doi:10.1371/journal.pcbi.1008272)
Supplement: Attachment — Submitted filename: Response_to_reviewer.docx [file pcbi.1008272.s007.docx]

**General answer to reviewers:**

We are thankful to the two reviewers and the Editor for the time invested and for their critical feedback. The quality of their comments helped us rephrase and clarify the manuscript.

Both reviewers were uncertain about the primary research question of the study. The primary research question of the study was to investigate where bumblebees would search for their home when confronted with a visual conflict indicating two possible home locations, and whether some well-known visually guided models could reproduce this behaviour. We have now re-written the complete introduction and conclusion to expose our research question clearly. In addition, the title of the paper has been changed.

As mentioned by the reviewers, only a selection of models with a limited set of parameter variations was analysed in our study. Therefore, we now explain more explicitly the reasons why we decided to investigate these particular models and no other ones. Furthermore, we discuss how alternative models could relate to our findings.

Firstly, we were interested in using holistic models over parameter-based models, because such models rely on a complete view of the environment without any *a priori* assumption about the existence of distinct landmarks. This makes holistic models more universal and parsimonious. Prior to our study, we questioned if holistic models will draw two catchment areas around the two cue-constellations as suggested by the behaviour, since they do not explicitly segregate cues. Interestingly these models predicted the complex conflict-dependent search behaviour of the bumblebees.

Secondly, among the tested snapshot-based models, the obvious parameter to be changed was the number of views. Consequently, we used models based on a different number of views. There is evidence that bees can use optic flow information to direct their foraging trip during local homing (Dittmar 2011). Therefore, we decided to test the same type of models with views based not only on brightness information as in previous studies, but also based on distance information. The selection of the different parameters (number of views, type of views) used by the selected models is now more clearly explained.

In the section below, we answer specific concerns of the two reviewers. We hope we understood the reviewers correctly and responded adequately to their concerns in our thoroughly revised manuscript.

The Authors,

**Reviewer #1:**
The authors trained bumblebees to navigate in a large cylindrical arena between the entrance of their nest (on the floor of the arena), and a feeder accessible through a small hole in the top part of the arena wall. The arena was designed to provide visual cues (3 stripes on the arena hole) and three cylindrical object standing on the floor around the nest entrance. The bumblebees homing search were analysed during tests were the two sets of cues were rotated relatively to each other, providing visual conflicts. In parallel, the authors implemented different visual-based homing models using reconstructed views of the arenas visual cues. Modelled agents were given some memories of the training situation, and tested in the different visual conflict situations. The authors calculated a fit (f1 score) for each model by comparing the likelihood where the agents were to end their journey (using Helmholtz-Hodge decomposition) to the spatial distribution of the bumblebees’ search, for each conflict situation. The model that obtained the highest overall fit (average f1 score across test situations) was equipped with a ‘Contrast-weighted Nearness’ visual front end views, that is, its visual input encodes both luminance and information about depth. The author concludes that encoding depth information (presumably through optic flow) form a ‘biologically plausible hypothesis’ (or ‘biologically inspired strategies’) to solve this kind of navigational tasks.

I first have to say that the bumblebee experiments, the models’ implementation, and the analysis are all quite ingenious, properly achieved and well described despite their necessary complexity. The figures are very neat and the results are well presented. This reflects a rare combination of high capability for both behavioural experiments and modelling.

I have however concerns about the more fundamental scientific approach used here.

It is not clear to me actually what is the scientific question here? Why did the authors choose to design these particular visual conflict situations? And why did they choose this 4 particular models with these specific set of parameters? The conclusions are based on the fit between the models and the bumblebees search, but it seems quite clear that this fit (and thus the conclusions) will be sensible to:

1- changing the parameters of the models (for instance, what if the number and position of the memorised views are different, what if the eyes are not spherical anymore? Etc..),

Indeed, the performance of a model is related to the position and number of views. However, we did not aim to compare the homing ability of different models systematically. But, solely trying to reproduce the search location of bees in conflict conditions. Nevertheless, we added details about the reason behind the choice of the views positioning, namely that bumblebees learning flights are in the beginning concentrated around the nest entrance. They loop around their nest incrementally increasing their distance from the nest-hole. Hence views taken within the cylinder constellation or close to the nest-hole are biologically plausible.

The question of the spherical eye was indeed not addressed as we rely on the models as described in the respective original study. The problem of non-omnidirectionality is interesting and should be addressed in a future dedicated study targeting both, the behavioural strategies employed by the animal and the modelling challenges.

2- changing the type of visual environment (for instance, an open vs cluttered natural scene would change the usefulness of depth information) or the type of visual conflict situation in this arena (what if the cylinders are removed?),

It is correct that our actual paradigm cannot and does not address the usefulness of depth information for homing.

The type of visual conflict tested in our experimental paradigm was explicitly designed to produce two plausible nest locations, thus creating an ambiguity. Of course, there might have been many other interesting possibilities to change the environment. However, these changes would have addressed a different question.

the way the bumblebee and model’s search is measured for comparison (what if, instead of ‘likelihood of ending up’, we look at the body orientation and straightness of paths ? Perhaps in that case the AVL (currently the poorest fit) would obtained the best fit, because to derive a direction the multiple snapshot models require to stop and scan at each time step (bumblebee don’t do that?) and the gradient descent or ascent require first to sample the neighbour locations (bumblebee don’t do that?).

We aimed at comparing model predictions and search locations of bees; we die not intend to analyse the method used by the agent to compare its memory and its actual view. Our research goal is now clearly discussed in the discussion part of the manuscript. Body orientation could not be resolved when filming the entire flight arena used in our study with only two cameras; in addition, the behaviours expressed by bees were quite heterogeneous when not searching. Hence the probability of ending up was a more robust metric in this paradigm.

Bumblebees indeed do not show scan and stop phases during return, or other apparent sampling behaviour.

3- the very choice of models (is there a reason for not testing a visual parameter based model (Möller 2001) for instance?).

   Möller, R. "Do insects use templates or parameters for landmark navigation?." Journal of Theoretical Biology 210.1 (2001): 33-45.

We answered this aspect in the general comment and manuscript.

If that is right, this makes your conclusion quite weak.


Currently, it feels like the modelled agents are used here as hypotheses themselves (which, as explained above, make the conclusions dependant on the specific parameter-agent-environment-measurements chosen), rather than as a tool to proof-test the viability of a specific hypothesis, or as a tool to explore the information available in environments. I exemplify the two latter approaches below.

The main conclusion here seems to be about the 'plausibility' and 'usefulness' of depth information, but these notions seems quite unrelated to the overall approach. If the question is about ‘the plausible use of depth through optic flow in bumblebee’, a definite answer can be obtained with a dedicated experiment, such as testing bees with landmarks that are camouflage against the background (as achieved previously by this lab in the past with bees (Dittmar et al. 2010)). Here a model can be used as proof-test that the camouflage landmark can be revealed using optic-flow as long as you do translational movement. If the question is about 'the usefulness of depth information for navigation in flying agents’, it can be tackled with a systematic analysis of the information provided by depth in natural scenes, and agents can be used to estimate the usefulness of this information for navigation (as achieved previously Stürzl, W. and Zeil, J. 2007).

Dittmar, L. et al. "Goal seeking in honeybees: matching of optic flow snapshots?." Journal of Experimental Biology 213.17 (2010): 2913-2923.

Stürzl, W. and Zeil, J. "Depth, contrast and view-based homing in outdoor scenes." Biological cybernetics 96.5 (2007): 519-531.
It is correct that our actual paradigm cannot and does not answer the relevance of depth information for homing. Indeed, as mentioned above, studies addressing this issue already exist and could justify the relevance of distance information via specific paradigms. In our study, we only aim to reproduce data in cue conflict situations. Because depth information as a mean to home has been shown and suggested by several studies, and because a model solely based on brightness could not account for some of the details of the behaviour, information about depth in the form of the contrast-weighted nearness was suggested as a rather simple alternative.

I would thus recommend to clearly establish a question together with hypotheses, and explain how the models and behavioural experiment will ‘a priori’ enable to disentangle the hypotheses and thus answer the question. Alternatively, it could be stated that the current study is not hypothesis driven but purely exploratory (but this may not be particularly helpful as such explorations are generally useful when achieved in settings were the observer wants to diminish the influence of its own assumptions) in that case it would require at least a detailed analysis of the bumblebees, and a detail analysis of models’ behaviour across their parameter space, and model should be chosen so as to embed general concept about either visual processing (what the nature of the visual information used (e.g., depth vs no depth)) or sensori-motor processing (how this information (whatever its nature) is transformed into action (e.g., gradient descent vs. multiple snapshot)), but not both at the same time.

Our study offers a systematic investigation of bumblebees’ search in an ambiguous scenario. The plus-value of our work is to be able to predict the bees’ specific behaviour, which suggests the segregation between the two cue constellations, with computationally parsimonious and holistic models. We could validate that holistic views are efficient to explain this behaviour, adding evidence that bees use such strategies during local homing.

The difference of performance between the models was more of a secondary aspect mostly used to assess how much minimalistic a model can be to reproduce the bees’ behaviour, i.e. having at least more than one view.

However, we do think the ability of these rather “simplistic” models to reproduce bumblebee behaviour is an interesting finding.

Finally, these findings are indeed linked to the assumptions made for the different models, but these assumptions are the ones often made to justify homing abilities of models, e.g. spherical eye, a memory of views, mental rotation. Nevertheless, these assumptions are now emphasised in the discussion.
I hope my comments will be perceived as constructive, and apologise if there has been misunderstanding on my side.
with best wishes,

**Reviewer #2: Comparative study of visually based homing models during visual conflict**

General Feedback:
I think this is an interesting study that combines new behavioural results with modelling analysis to advance our understanding of how insects pinpoint inconspicuous locations like a small nest entrance. As such it is well suited to the readership of PloS Comp Bio.

I have no major concerns about validity of the behavioural or modelling works but think that the paper needs some series editing before I can recommend for publication in PloS Comp Bio. Below I list my main concerns and where I feel it appropriate offer some opinions on how they might be addressed.

Introduction:
Here is where I have some of my most series issues with the current manuscript.

Firstly sufficient care has not been taken to cite original papers. For example, for route following in ants you should be citing Kohler and Wehner, 2005 and Mangan and Webb, 2012 rather than paper you currently cite which instead speaks about temporal information for place recognition (Graham and Mangan, 2015). There are other examples.
Secondly, if you are to perform a comparative study of models there is a clear need to (a) provide comprehensive coverage of what models, or at least model categories, have been proposed and their strengths and weaknesses. This is currently lacking with many models missing e.g. from Jochen Zeil, Wolfgang Sturzl and recent models using frequency cues e.g. Stone et al, 2018 "Rotation invariant visual processing for spatial memory in insects". In this regard, there have been a number of comparative studies on visual homing implemented previously which are missing from the current text e.g. Vardy A (2005) Biologically plausible methods for robot visual homing. PhD thesis, Ottawa-Carleton Institute for Computer Science"; Basten Mangan and Webb, 2009 Modelling place memory in crickets; and even Wystrach et al, 2013 "Snapshots in ants? New interpretations of paradigmatic experiments". Indeed, Jochen Zeil's 2012 paper: "Insect Homing: an insect perspective" is highly relevant here. Note, on the above comment, the research of Jochen Zeil focusses on the final approach of digger wasps to their inconspicuous nest entrance and appears particularly relevant here. I recommend a thorough redraft of the literature review to set the context for the reader.

We now reformatted the introduction and discussion, to include missing references.

This leads directly to my next concern. What motivated your choice of model to implement and test? This is not clear to me from the current paper. If this is to be a truly comparative study, then you should be testing across a range of models, or model categories, that give a good representation of the current hypotheses from the field. Otherwise, it can appear as a straw man argument to support your preferred model. Note: I am not suggesting that you reimplement models to add to your data but instead describe your criteria for model selection and then discuss how your results gathered on a sample of models impact current theories.

Our main interest was to look where bumblebees will search for their nest when there exist two possible nest locations and second find a suitable model which could predict their search location in such an ambiguous situation. We rephrased the introduction to make the research question clearer. Numerous variants of homing models have been described and tested in the last decades. Still, we sought first for computationally parsimonious models to predict the bumblebees’ search location in visual conflict situations. We only wanted to increase the computational complexity of the models if none of the simpler models would be able to explain the data. Also, implications with current theories/models are now developed in the discussion.

If I may offer some advice it seems to me that you are not focussed enough on testing a specific hypothesis. I would suggest that two recent hypotheses arise from the works of Dewar (as you refer to later in your paper) who proposes that by aligning with multi nest facing views one can return home, and Sturzl et al, 2016; Le Moel, 2020 that propose instead having mutliple repulsive view leads to more robust homing. In essence, your study is testing the validity of Dewar's proposal using a visual distortion (not a cue conflict scenario) paradigm and your results support his work. Thus, I would restructure the Intro to focus on this key issue and work from there.

Dewar et al. 2014 proposed a model using only attractive views, thus implying the agent to physically rotate to scan the environment or to rotate its view mentally. Kodzhabashev & Mangan 2015, suggested a model based on an oscillating mechanism and familiarity to follow a route. However, for homing this model may not be appropriate as it was shown by Le Möel 2020. Adding repulsive views enable an agent using an oscillator, to home. The main difference between Dewar and Le Möel is the mechanism allowing the agent to sample the visual information about the environment, i.e. by oscillating or rotating. This question is ecologically relevant; therefore, we now discuss its implication in the discussion.

The term “conflict-scenario” is now often replaced by “ambiguous situation”, however, this term is still used mainly in the manuscript. In fact, the “conflict-angle” between the cues is a clearer nomination to explain the different tested conditions.

.

Behavioural Study:
The data presented gives n=4 example flights but then search distributions for n=5-10 flights. What I am not sure about, and cannot find explained in the manuscript is how this data is made e.g. is this one track from 4 different bees or multiple tracks from the same bees. This is very important and should be clearly stated.

This is a really important remark, which is now explained in a full paragraph in the method section.

I think the search patterns shown are an excellent way to show the data but I think Fig 4 could do with more labelling and also some additional clarity. For example, the reader has to constantly look back to A to recall where the landmarks were in training to compare with the new results. Also, they need to decode what each title means. You could add an outer ring to each figure showing the location of landmarks in training and a labelled arrow showing that rotation. Similarly, you could show the location of the landmarks as white dots in each figure and a similarly labelled arrow to show that rotation. That would make it easier for readers to understand.

The figure has been updated to show the different cue displacements following the above suggestions (arrows outside the arena top view). The sub-titles are now explained in the added procedure paragraph.

Modelling study:
For the final approach, there are two models describing how views could be used in a 'repulsive' manner to drive approach. "W Stürzl - ‎2016, How wasps acquire and use views for homing"; "" Opposing views method. Le Möel, 2020 "Opponent processes in visual memories: A model of attraction and repulsion in navigating insects’ mushroom bodies". How would this compare?

Le Möel 2020 was not published at the submission date (2019), hence at first not included. But because of its relevance, we now discussed its possible implication for our present study.

The study of Stürzl et al. 2016, focuses on the wasps’ last approach to the nest, and how their subtle flight characteristics match the guidance based on views collected upon learning. Unfortunately, precise body orientation could not be extracted from our trajectory, thus not allowing us to compare our results with those of the study of Stürzl et al. (2016).

Do you not have data on the learning flights which they could add to the models? Are these structured that make one model in particular better? I think adding this element to the modelling work would be very interesting and increase the impact a lot.
Addition of learning flights would indeed be interesting. However, from several previous studies dedicated explicitly on bumblebee learning flights (Philippides 2013, Lobecke 2018) it has not yet been possible to infer from their choreography when and where snapshots would be collected. Nevertheless, we believe the assumptions made about the location of the collected views is biologically plausible, and we explain in the manuscript the motivation of the placement of the view.

Why include the results for the ALV at all? This model has already been shown unable to account for animal data numerous times and just confuses the matter. The benefits of multi views is for me the main contribution. I am not sure that the reader gains anything from the inclusion of the ALV data.

The ALV is selected since it is a well-established, often studied and computationally cheap homing model offering an alternative to snapshot navigation. In addition, its results notably for the F1-score, can be used as a reference, since this model is failing at reproducing the behaviour.

Specific Comments

Terminology - there is repeated use of the phrase "to find back home" which is strange in English. I would replace with either "to return home", or to "to find home"

This has been changed.

Table 1. should memory for CWN multi-snapshot not be "several CwN snapshots"?
